# The TLR5 Agonist Flagellin Shapes Phenotypical and Functional Activation of Lung Mucosal Antigen Presenting Cells in Neonatal Mice

**DOI:** 10.3389/fimmu.2020.00171

**Published:** 2020-02-18

**Authors:** Pankaj Sharma, Ofer Levy, David J. Dowling

**Affiliations:** ^1^Precision Vaccines Program, Division of Infectious Diseases, Boston Children's Hospital, Boston, MA, United States; ^2^Harvard Medical School, Boston, MA, United States; ^3^Broad Institute of MIT and Harvard, Cambridge, MA, United States

**Keywords:** early life immunization, newborn, dendritic cells, mucosal immunity, cross presentation, TLR5, flagellin

## Abstract

Intranasal mucosal vaccines are an attractive approach to induce protective mucosal immune responses. Activation of lung antigen presenting cells (APCs), a phenotypically and functionally heterogeneous cell population located at distinct mucosal sites, may be key to the immunogenicity of such vaccines. Understanding responsiveness of newborn lung APCs to adjuvants may the inform design of efficacious intranasal vaccines for early life, when most infections occur. Here, we characterized and phenotyped APCs from neonatal (7 days of life) and adult (6–8 weeks of age) mice. Neonatal mice demonstrated a relatively high abundance of alveolar macrophages (AMs), with lower percentages of plasmacytoid dendritic cells (pDCs), CD103^+^ (cDC1), and CD11b^+^ (cDC2) DCs. Furthermore, neonatal CD103^+^ and CD11b^+^ DC subsets demonstrated a significantly lower expression of maturation markers (CD40, CD80, and CD86) as compared to adult mice. Upon stimulation of lung APC subsets with a panel of pattern recognition receptor (PRR) agonists, including those engaging TLRs or STING, CD11c^+^ enriched cells from neonatal and adult mice lungs demonstrated distinct maturation profiles. Of the agonists tested, the TLR5 ligand, flagellin, was most effective at activating neonatal lung APCs, inducing significantly higher expression of maturation markers on CD103^+^ (cDC1) and CD11b^+^ (cDC2) subsets. Intranasal administration of flagellin induced a distinct migration of CD103^+^ and CD11b^+^ DC subsets to the mediastinal lymph nodes (mLNs) of neonatal mice. Overall, these findings highlight age-specific differences in the maturation and responsiveness of lung APC subsets to different PRR agonists. The unique efficacy of flagellin in enhancing lung APC activity suggests that it may serve as an effective adjuvant for early life mucosal vaccines.

## Introduction

The persistently high global burden of infections in the very young provides a compelling rationale for developing additional safe and effective early life vaccines (1). Most childhood pathogens access the body through mucosal membranes (2). Additionally, viral infections including respiratory syncytial virus (RSV) and influenza virus are often more severe and/or prolonged in early life as compared to adult life (3). Parenteral vaccines, such as those delivered intramuscularly, are often poor inducers of protective immunity at mucosal surfaces (4). While, effective immunization against mucosal infections usually requires topical-mucosal vaccine administration, which could neutralize the pathogen on the mucosal surface before it can cause infection, only a few human vaccines are oral- e.g., those against cholera, typhoid, polio, and rotavirus, and only one, an influenza vaccine, is administered intranasally (5). Moreover, vaccines delivered via different routes interact with different APCs that activate distinct arms of immunity (6). Tissue distribution and migratory properties of APCs, such as alveolar macrophages (AMs), plasmacytoid DCs (pDCs), and conventional DCs (cDCs) contribute to the generation of distinct immune responses (7). In mice, cDCs have been classified into two major subsets, cDC1, which express CD103 (CD8α) and specialize in cross-presentation to CD8^+^ T cells critical for immunity against intracellular pathogens, viruses, and cancer; and cDC2, which express CD11b and promote CD4^+^ T cell differentiation into subsets specializing in anti-viral, -fungal, or -helminth immunity (8). Immune cells in the neonatal lung differ in quantity and quality from adults and, hence, react differently to environmental, microbial, and vaccine exposures (9). Mucosal administration of antigen may drive a more effective mucosal response to respiratory infections (10, 11), potentially reflecting activation of antigen-specific secretory IgA responses and development of lung resident memory T cells (12–14).

Generation of mucosal immunity may be achieved via various routes including oral, intranasal (pulmonary), rectal, and vaginal (4). The intranasal route is attractive considering injection-free delivery (15), and ease of clinical administration (16) in early life. However, the challenge to early life intranasal vaccination can be the induction of non-specific inflammation and/or the generation of tolerance to unknown or novel antigens (17). Such challenges may be a particular concern in early life, where the T-helper response is biased toward type-2 immunity, exacerbation of which may predispose individuals to eosinophilia and pulmonary disorders (18). Nevertheless, solutions to such challenges may be found in the use of precision adjuvants (19) which drive the appropriate activation of APCs and help in shaping the immune system (20, 21). Adjuvantation is a key tool to enhance vaccine-induced immunity (22). Adjuvants can enhance, prolong, and modulate immune responses to vaccine antigens to maximize protective immunity (21, 22), and may potentially enable more effective immunization in the very young and the elderly (23). Despite evidence suggesting ontological differences in the innate response in the mucosal APCs, there are gaps in the understanding of basic mechanisms and whether adjuvants could drive mature and enhance the functions of lung APC subsets in diverse age groups such as infant and neonates (24). A deeper understanding of mucosal APCs in early life may inform the design of effective mucosal vaccines for the very young.

In the present study, we examined the phenotypic and functional differences in mucosal APC subsets isolated from newborn and adult murine lungs and characterized their responsiveness to different PRR agonists/adjuvants. We found that DC subsets from neonate mice lungs are phenotypically and functionally distinct from those of the adult mice, as they exhibit a different activation pattern to various PRR agonists. The TLR5 agonist flagellin, a globular protein that arranges itself in a hollow cylinder to form the filament in a bacterial flagellum, strongly activated lung migratory DC (migDCs) subsets and upregulated their expression of CD40, CD80, CD86, and CCR7. Also, when used as an adjuvant during intranasal vaccination, flagellin regulated the migration of DC subsets to the draining lymph nodes, and potentiated the phagosome maturation in CD103^+^ DC subset, correlating with their ability to mount a robust antigen cross presentation phenontype. These findings suggest that TLR5 may play an important role in the maturation and activation of lung migratory DCs in neonates and could be an empirically promising target for intranasal mucosal vaccines delivered in early life.

## Materials and Methods

### Animals

All experiments involving animals were approved by the Animal Care and Use Committee of Boston Children's Hospital and Harvard Medical School (protocol numbers 15-11-3011 and 16-02-3130). C57BL/6 mice were obtained from Taconic Biosciences or Charles River Laboratories and housed in specific pathogen-free conditions in the animal research facilities at Boston Children's Hospital. For breeding purposes, mice were housed in couples, and cages checked daily to assess pregnancy status of dams and/or the presence of pups. When a new litter was discovered, that day was recorded as day of life (DOL) 0. Both male and female pups were used for experiments. CO_2_ was used as the primary euthanization method, with cervical dislocation as a secondary physical method to ensure death.

### Reagents

Collagenase-1, DNase-1, 3-(4,5- dimethylthiazol-2-yl)-2,5-diphenyltetrazolium bromide (MTT), Griess reagent, FITC-dextran, and Concanavalin-A were all purchased from Sigma Aldrich (St. Louis, MO). CD11c magnetic cell separation kit (MACS) were purchased from Miltenyi Biotec (Bergisch-Gladbach, Germany). All monoclonal antibodies (Abs) were purchased from eBioscience (San Diego, CA), while all the PRR agonists were purchased from *InvivoGen* (San Diego, CA). For all *in vivo* studies, ultrapure *Salmonella typhimurium* flagellin (FLA-ST) from *InvivoGen* (endotoxin level < 0.05 endotoxin units (EU)/mg) and was employed. FLA-ST is purified by acid hydrolysis, heating and ultrafiltration to obtain an estimated at 10% purity, before an additional purification step using monoclonal anti-flagellin affinity chromatography to obtain purity >95%.

### Preparation of Single Cell Suspension From Murine Lungs and Isolation of Lung APCs

To prepare single cell suspensions, neonates and adult mice were euthanized and the lungs were exposed by dissecting through the thorax by raising the sternum to avoid any injury to the lung. Mice lungs were perfused through the right ventricle by adding ice cold Phosphate-Buffered Saline (PBS) [containing 2 mM ethylenediaminetetraacetic acid (EDTA)] and the lung lobes were carefully isolated and manually minced into small pieces in petri dishes. Next, these minced pieces were incubated at 37°C for 30 min in a digestion medium containing 2 mg/ml of collagenase and 80 U/ml of DNase-1. Digested lung tissue was subsequently passed through a 40 μM cell strainer and centrifuged at 300 g for 10 min at 4°C. Red blood cell (RBC) lysis was carried out in the cell pellet using BD Pharm Lyse following instructions of the manufacturer (*BD Biosciences*). For multicolor immunophenotyping and *in vitro* stimulation assay, lung APCs were enriched using CD11c based magnetic activated cell sorting (MACS) following the manufacturer's instructions.

### *In vitro* Stimulation Assay

All PRR agonists employed in the study were verified endotoxin-free as indicated by the manufacturers (*InvivoGen*). For stimulation experiments, isolated lung CD11c^+^ cells from newborn and adult mice were plated in round bottom 96-wells non-tissue culture-treated plates at the density of 10^5^ cells/well in 200 μl of fresh complete culture medium and stimulated for 24 h with 100 ng/ml of Pam3CysSerLys4 (PAM3CSK4), PAM2CSK4, Poly I:C, Monophosphoryl Lipid A (MPLA), *S. typhimurium* flagellin, 5′ppp-dsRNA; 0.1 μM of CL075, CpG class C—oligonucleotides (ODN) 2,395 or 100 μg/ml of cyclic [G(2′,5′)pA(3′,5′)p] (2′3′-cGAMP, hereto referred to as cGAMP). Stimulation concentrations were chosen based on optimal maturation responses achieved (cytokine production, upregulation of co-stimulatory markers) in murine neonatal and adult bone-marrow dendritic cell assays, as previously described by the authors (25, 26). Cultured DCs demonstrated no obvious signs of spontaneous maturation in the control conditions.

### Flow Cytometry

CD11c enriched cells from mice lungs were stained with the monoclonal Abs directed against CD11c, CD11b, CD103, F4/80, and plasmacytoid dendritic cell antigen-1 [PDCA-1 (also known as CD317)] to identify the five major APC populations in the lung. To monitor the expression of co-stimulatory molecules, *in vitro* stimulated CD11c^+^ cells were washed with ice cold PBS once and stained with Ab cocktail containing monoclonal Abs for CD40, CD80, and CD86 along with APC surface markers. Data were acquired on BD Fortessa flow cytometer and was analyzed using Flowjo (Treestar). All Abs used for flow cytometry are listed in Table S1.

### Analysis of Phagocytosis and Antigen Processing Capacity of Lung APCs

Isolated CD11c^+^ cells were incubated with either FITC-dextran (1 mg/ml) or with DQ™ ovalbumin (DQ-OVA) (0.5 mg/ml) for 45 min in a CO_2_ incubator at 37°C. The fluorescence of gated cells was measured using BD Fortessa as described recently (27).

### Intranasal Administration of DQ-OVA

Fifty microliters of PBS, DQ-OVA (50 μg), and flagellin (3 μg total) adjuvanted DQ-OVA (50 μg) were applied *via* the nostrils as recently described (28). DQ-OVA was used for degradation and accumulation assays as it consists of OVA bound to a self-quenching fluorescent dye, which upon intracellular degradation releases specific fluorescence (excitation at 505 nm, emission at 515 nm). Accumulated DQ-OVA forming dimers emit fluorescence in a different channel (excitation at 488 nm, emission at 613 nm). Animals were euthanized 4 or 24 h after intranasal administration and lung and or lung draining lymph nodes were harvested to determine the uptake, trafficking, phenotype, and antigen degradation (28). Tracking the quantity and localization of specific APC *in vivo* was achieved via the use of a SIINFEKL monoclonal Ab (mAb), which specifically reacts with ovalbumin-derived peptide SIINFEKL bound to H-2Kb of major histocompatibility complex (MHC) class I. To enumerate the antigen cross presentation, DCs from the draining lymph nodes were stained with PE anti-mouse MHC class I molecule (Kb) bound to the peptide SIINFEKL Abs (Kb-SIINFEKL) (*BioLegend*; San Diego, CA). Data was acquired on BD Fortessa flow cytometer.

### Statistical Analysis

Statistical significance and graphs were generated using Prism v. 7.0a (GraphPad Software, La Jolla, CA, USA) and Microsoft Excel (Microsoft Corporation, Redmond, WA). For data analyzed by normalization to control values (vehicle), column statistics were conducted using the two-tailed Wilcoxon Signed Rank Test or unpaired Mann-Whitney test as appropriate. Group comparisons employed two-way Analysis of variance (ANOVA) with Sidak multiple comparison post-test. Results were considered significant at *p* < 0.05, and indicated as follows: *p < 0.05, **p < 0.01, ***p < 0.001, ****p < 0.001.

## Results

### Neonate Murine Lung Antigen Presenting Cells and Their Precursors Demonstrate Distinct Percentages and Co-stimulatory Molecule Expression

**Figure 1:**
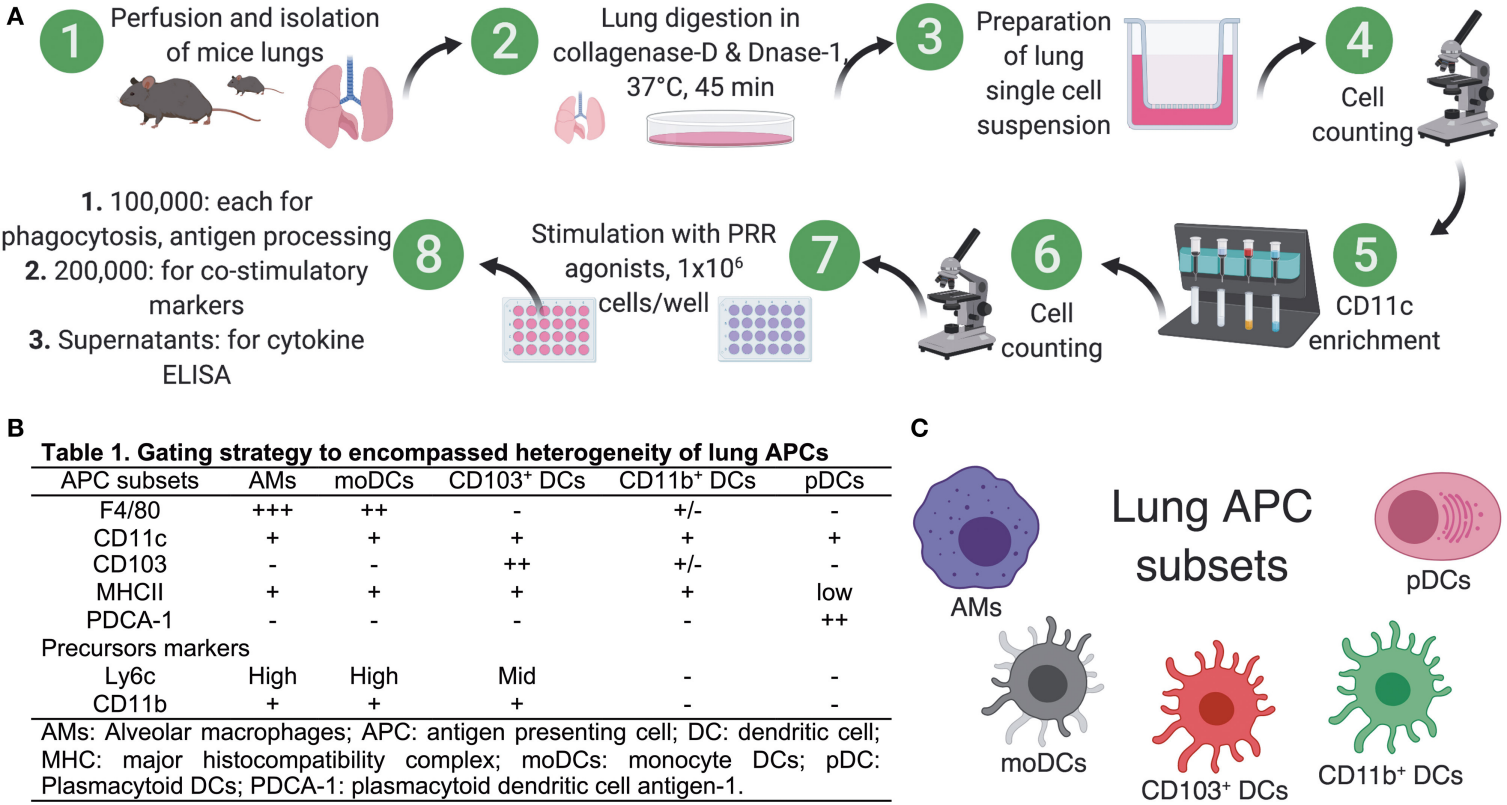
Preparation of single cell suspensions from neonatal and adult murine lungs for isolation of APCs. **(A)** To prepare single cell suspensions, neonatal and adult mice were euthanized and the lungs were exposed by dissecting through the thorax by raising the sternum to avoid any injury to the lung, perfused through right ventricle by adding ice cold PBS (containing 2 mM EDTA) and lung lobes were carefully isolated and were manually minced into small pieces in Petri dishes. Minced pieces were digested and digested lung tissue was subsequently passed through a cell strainer. For multicolor immunophenotyping and *in vitro* stimulation assay, lung APCs were enriched using CD11c- based magnetic beads for use in *in vitro* stimulation assays. **(B)** List of known pan- and precursor- markers used in combination to identify heterogeneous populations of lung APCs. **(C)** Overview of the lung APCs studied in this study.

**Figure 2:**
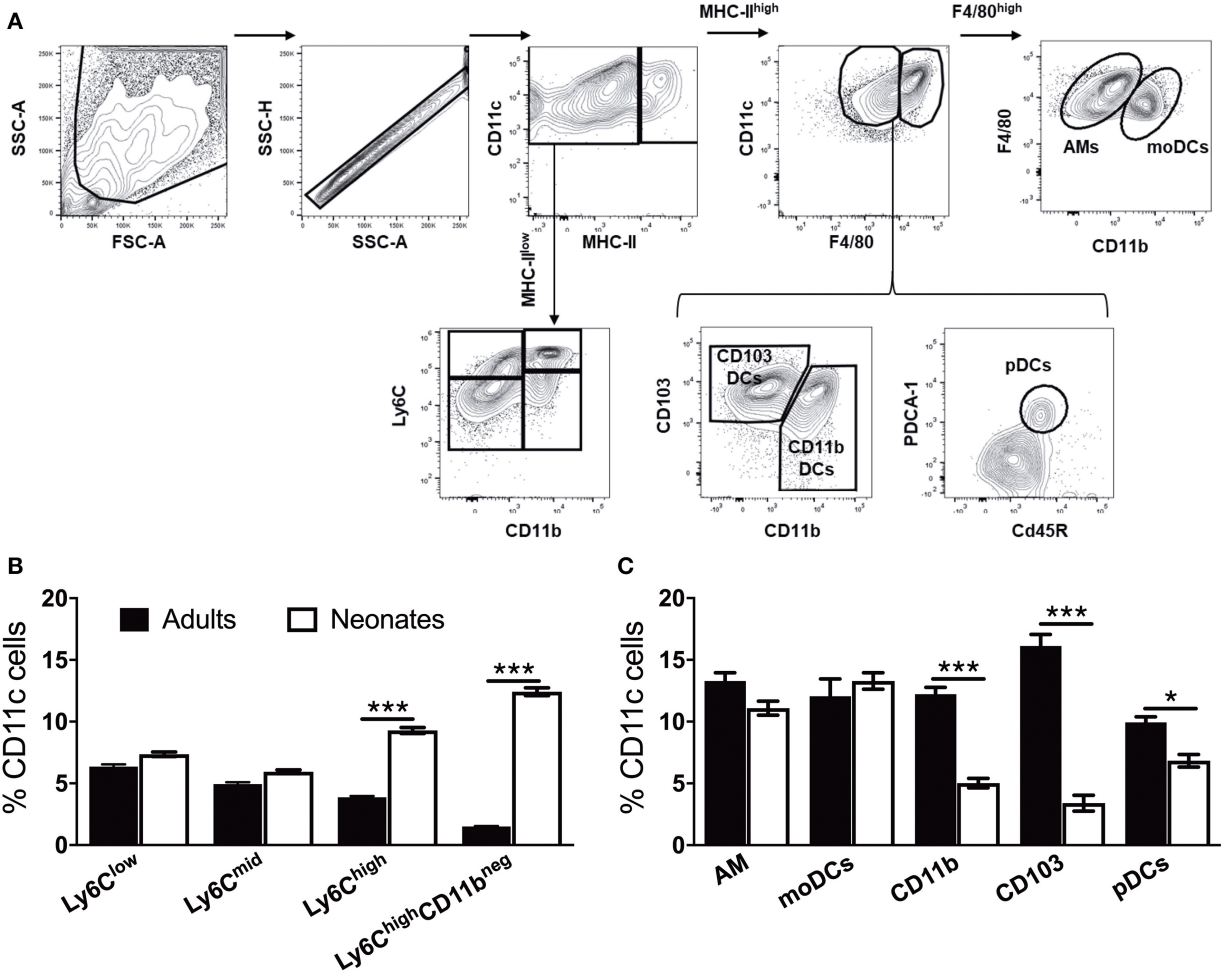
Neonate murine lung antigen presenting cells and their precursors demonstrate distinct subset percentages. **(A)** An overview of the flow cytometry gating strategy used for the identification of murine APC precursors and subsets in both adult and neonatal lungs. **(B)** The percentage of APC precursors, as defined by CD11c, Ly6C, and CD11b cell expression. **(C)** The percentage of APC subsets, as defined by CD11c, F4/80, CD11b, CD103 PDCA-1, and CD45R cell expression in mice lungs. Results are expressed as mean ± SEM of *n* = 12 per age group. *p < 0.05, ***p < 0.001 determined by repeated measures two-way ANOVA with Sidak *post-hoc* test.

**Figure 3:**
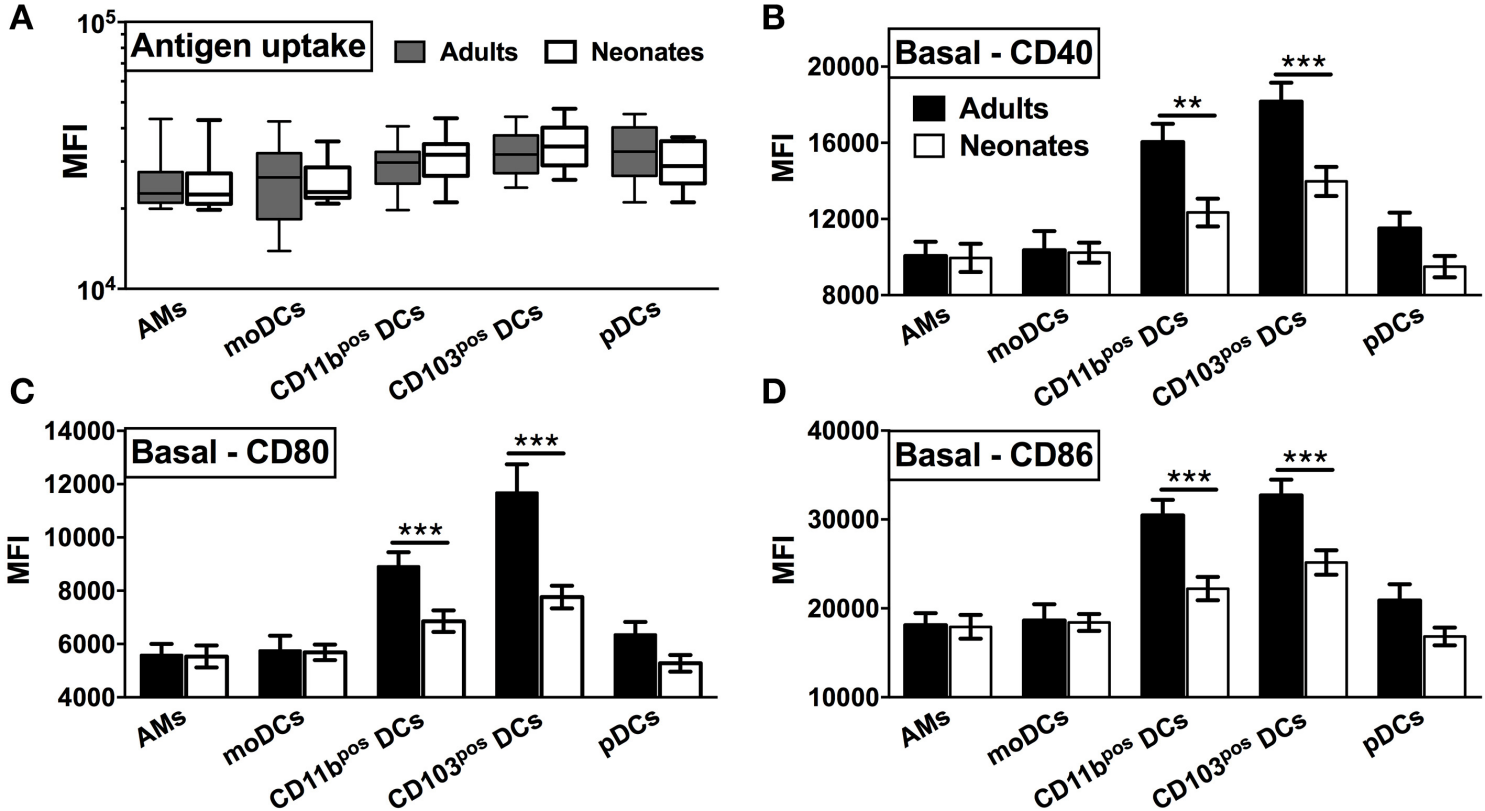
Neonatal murine lung CD103^+^ DCs and CD11b^+^ DC subsets demonstrate distinct basal co-stimulatory molecule expression. **(A)** Similar antigen uptake capacity of neonatal and adult APCs was noted across all the DC subsets. **(B–D)** Neonatal CD103^+^ DCs and CD11b^+^ lung DC subsets demonstrate a lower expression of co-stimulatory molecules CD40, CD80, and CD86 as compared to adult mice. Results are expressed as mean ± SEM of *n* = 12 per age group. **p < 0.01, ***p < 0.001 determined by repeated measures two-way ANOVA with Sidak *post-hoc test*.

To prepare single cell suspensions, neonates and adult mice were euthanized and the lungs were isolated, digested, and enriched for CD11c^+^ cell fractions (Figure 1A). A panel of cell surface makers were used in combination to identify heterogeneous populations of lung APCs studied in this article (Figures 1B,C). As mucosal APCs are heterogeneous, we employed a stringent gating strategy to distinguish the different subsets and their progenitors. By using the combination of different surface markers and a hierarchal gating strategy, we identified alveolar macrophages (AMs), moDCs, CD103^+^ DCs, CD11b^+^ DCs, and pDCs (Figure 2A). While the percentage of Ly6C^+^ cells was significantly higher in neonatal vs. adult mice (Figure 2B), and AMs and moDCs subsets were similar between both age groups, neonates demonstrated a significanly lower percentage of lung DC subsets CD103^+^ DCs (*n* = 12, *P* < 0.001), CD11b^+^ DCs (*P* < 0.001), and pDCs (*P* < 0.05) (Figure 2C). Remarkably, while both adult and neonatal antigen uptake capability was similar for all the major APC subsets (Figure 3A), CD103^+^ and CD11b^+^ DCs from neonates demonstrated relatively lower expression of basal co-stimulatory molecules CD40 [CD11b^+^ (*n* = 12, *P* < 0.001) and CD103^+^ (*P* < 0.01) DCs], CD80 (*P* < 0.001), and CD86 (*P* < 0.001) (Figures 3B–D). These observations demonstrated that neonatal murine lung APCs contain a greater proportion of phenotypically distinct immature DC precursor cells, including CD103^+^ and CD11b^+^ DCs, relative to their adult counterparts.

### Neonatal Murine Lung APCs Demonstrate Distinct Functional Responses to PRR Agonists

**Figure 4:**
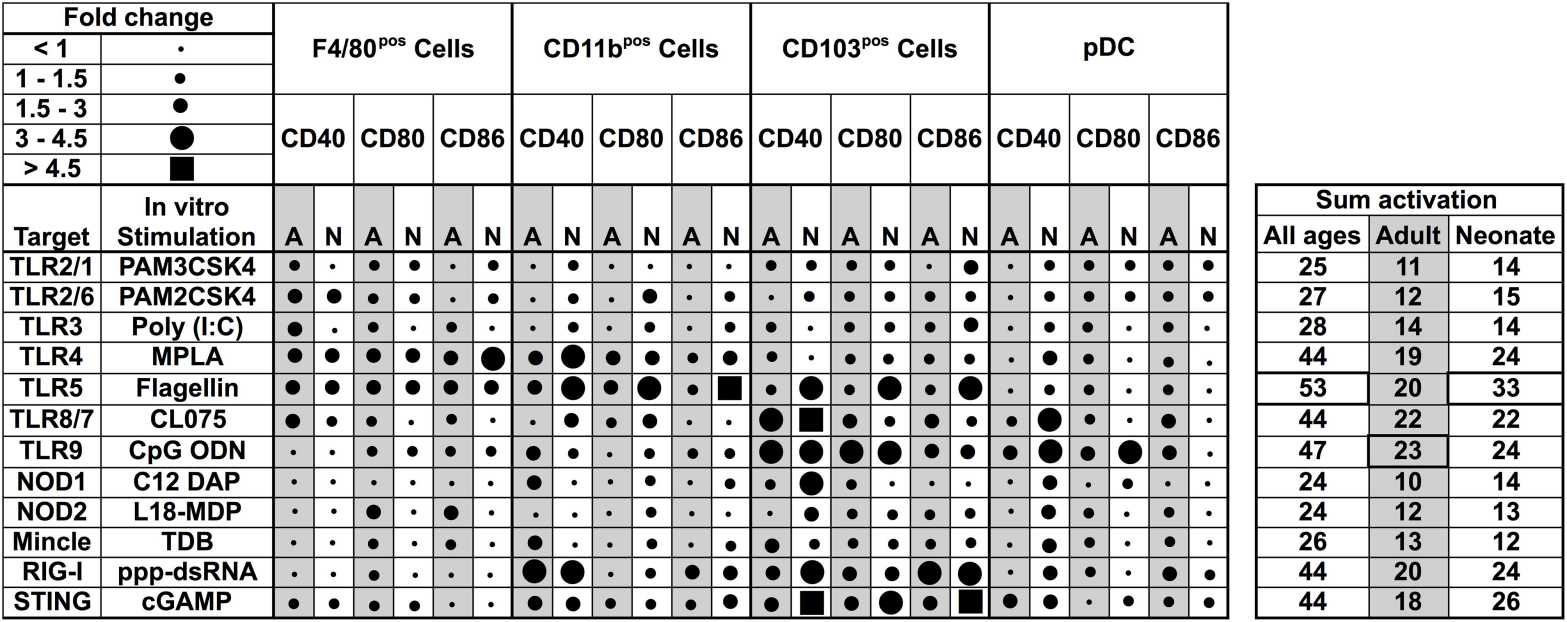
Functional differences in the major APC subsets in mice lungs identify flagellin and 2′3′-cGAMP as potential activators of mucosal immunity in early life. Summary of flow cytometry all APCs maturation data collected *ex vivo* stimulation. Dot sizes indicate the fold-change (ranging from <1 to >4.5) for CD40, CD80, and CD86, per F4/80, CD11b, CD103 PDCA-1 APC subsets, as compared with un-stimulated controls per age group. Gray-shaded sections indicate adult data. Combined activation total fold-changes are summarized in the right panel. Upon stimulation with PRR agonists TLR2/1 (PAM3CSK4), TLR2/6 (PAM2CSK4), TLR3 (Poly I:C), NOD1, and NOD2 ligands (C12-iE-DAP and L18-MDP, respectively), APC subsets were moderately activated. Contrary to these ligands, the TLR5 agonist flagellin and STING agonist 2′3′-cGAMP specifically and robustly activated neonatal CD103^+^ and CD11b^+^ DC subsets, while the TLR4, TLR8/7, TLR9, and RIG-I (5′ppp-dsRNA) demonstrated comparable activity for all the DC subsets from the neonates and adult mice. *n* = 5 per age group.

Our observations regarding the phenotype of DC subsets in neonatal murine lungs indicated an intrinsic age-specific difference in the percentages and maturation status of migratory APC subsets, raising the possibility that functional responses of migDCs subsets to stimuli such as PRR agonist adjuvants may also be distinct. To test this hypothesis, we stimulated CD11c^+^ cells isolated from the lungs of different aged mice and stimulated them with different classes of PRR receptor agonists at a concentrations reported most active for bone marrow DC activation (25, 26). We then summarized the combined flow cytometry all APCs maturation data collected following *ex vivo* stimulation, and graphed the data as volumetric dot sizes indicating the relative fold change (ranging from <1 to >4.5) for CD40, CD80 and CD86, per F4/80, CD11b, CD103, PDCA-1 APC subsets, as compared with un-stimulated controls per age group. Overall, unique activation and maturation patterns were observed by stimuli, age, marker, and APC subset (Figure 4). Ranking by sum fold-change activation, CpG ODN ranked first in adult mice. Flagellin ranked first in the ability to mature neonatal lung APCs (Figure 4). PRR agonists TLR1 (PAM3CSK4), TLR2 (PAM2CSK4), and TLR3 (Poly I:C), NOD1 (C12-iE-DAP) and NOD2 (and L18-MDP) moderately activated APCs from neonatal adult mice lungs. Contrary to these ligands, the TLR5 agonist flagellin and STING agonist 2′3′-cGAMP specifically and strongly activated the neonatal CD103^+^ and CD11b^+^ DC subsets, while the TLR4 (MPLA), TLR8/7 (CL075), TLR9 (CpG), and RIG-I (5′ppp-dsRNA) showed comparable activity for all the DC subsets from the neonates and adult mice. We also assessed cytokine production by lung CD11c enriched cells after stimulation. As compared to adults, APCs from neonatal mice produced lower IL-12p70, IL-6, TNF, and IL-1β in responses to NOD1, TLR2/6, 3, 4, 8/7, and 9 stimulation, while TLR5 (flagellin), RIG-I (5′ppp-dsRNA) and STING (2′3′-cGAMP) stimulation mounted a comparable cytokine response from neonatal and adult APCs (Figure S1). These observations demonstrate functional differences in the major APC subsets of murine lungs and highlight flagellin and 2′3′-cGAMP as potential activators of mucosal immunity in early life.

### Flagellin Upregulates the Expression of CCR7 on Neonatal CD11c Enriched Cells and Drives the Lymph Node Homing of migDC Subsets

**Figure 5:**
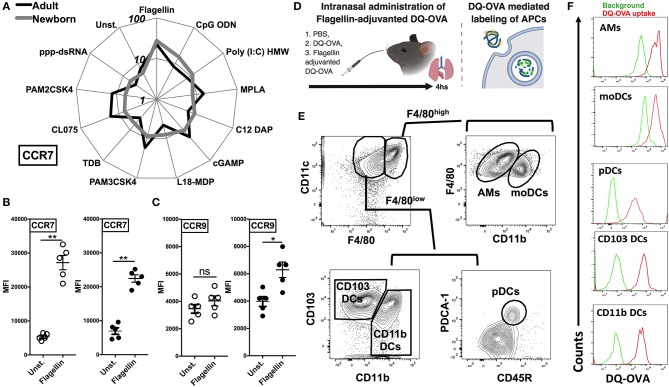
Flagellin upregulates the expression of CCR7 on neonatal CD11c enriched cells and drives the lymph node homing of migDC subsets. **(A)** Characterization of lung CD11c^+^ cells isolated from the neonatal and adult mice, identifies TLR5 agonist flagellin as the most robust inducer of the expression of lymph node homing receptor CCR7 [MFI ranked clockwise high (flagellin) to low (unstimulated)]. **(B,C)** Flagellin induced CCR7 expression is inversely related to the expression of CCR9 on neonatal lung CD11c^+^ DCs. **(D)** Overview of Intranasal administration of Flagellin-adjuvanted DQ-OVA and DQ-OVA mediated labeling of APCs. DQ-OVA is a self-quenched conjugate of ovalbumin antigen that exhibits bright green fluorescence signal only upon proteolytic cleavage/degradation by endosomal proteases. **(E)** Representative gating strategy for identification of APC subsets in adult mice lungs. **(F)** DQ-OVA uptake by adult lung APC subsets 4 h after intranasal administration. *n* = 5 per age group. For analyses of individual treatments (e.g., unstimulated vs. flagellin), the unpaired Mann-Whitney test was applied at each concentration, and statistical significance denoted as *p < 0.05, **p < 0.01.

As different stimuli induce distinct activation of lung APC subsets, we characterized the expression of lymph node homing receptor CCR7 on lung CD11c^+^ cells isolated from the neonatal and adult mice. CCR7 and CCR9 are both chemokine receptors that regulate homing of immune cells. Specifically, up-regulation of CCR7 on mature DCs is required for migration to draining/secondary lymphoid organs (29, 30). Conversely, immature DCs express high levels of CCR9, as an indication of lower migration potential (31). Consistent with the *in vitro* co-stimulatory molecule stimulation data, and when ranked by MFI, MPLA, CL075, CpG-ODN, and STING demonstrated moderate upregulation of CCR7 on neonates as well as on the adult APCs (Figure 5A), while as compared to unstimulated controls, flagellin most strongly upregulated the expression of CCR7 on neonatal APCs (Figure 5B, *P* < 0.001) and to a significant but lesser level in adults (*P* < 0.01). Conversely, as compared to adults (*P* < 0.05), neonatal lung CD11c^+^ DCs CCR9 expression was not significantly increased (Figure 5C). We also noted that the APCs from the neonates and adults responded similarly to the NOD1 and NOD2 agonists studied (C12-iE-DAP and L18-MDP) (Figures 4, 5A). These findings suggest that TLR5 might drive the migration of DC subsets to draining lymph nodes and initiate adaptive immunity.

To test this hypothesis, we administrated the DQ-OVA antigen either alone or in combination with flagellin by intranasal route and measured migration of lung DCs to draining lymph nodes (Figure 5D). DQ-OVA is a self-quenched conjugate of ovalbumin antigen that exhibits bright green fluorescence signal only upon proteolytic cleavage/degradation by endosomal proteases (32) (Figure 5D). Since, as we outlined in Figure 2A, DC subsets from neonates and adult showed no significant differences in the antigen uptake, we could rely on DQ-OVA mediated labeling for these studies. Also, to rule out any difference which might be because of accessibility of DQ-OVA to lung interstitial resident DCs (moDCs and CD11b DCs), we looked at DQ-OVA uptake by lung APC subsets 4 h after intranasal administration. Our data clearly demonstrated that all the major APC subsets were labeled (Figures 5E,F).

**Figure 6:**
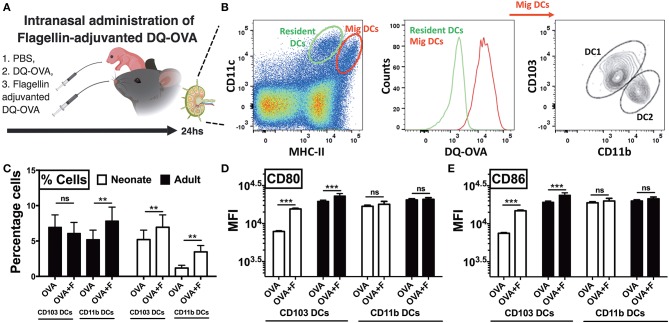
Neonatal mice demonstrate polarized migration of CD103^+^ and CD11b^+^ DC subsets to the mediastinal lymph nodes (mLNs) after intranasal administration of flagellin. **(A)** Overview of Intranasal administration of flagellin-adjuvanted DQ-OVA and isolation of draining lymph nodes at 24 h. **(B)** Gating strategy for identification of migratory DC1s and DC2s in draining lymph nodes. **(C)** Flagellin significantly increases the percentage of OVA-specific CD11b^+^ cells in mLNs of adult mice, but not CD103^+^ DCs. Flagellin significantly increases the percentage of both OVA-specific CD103^+^ and CD11b^+^ DC subsets in neonates. **(D,E)** Flagellin upregulates expression of CD80 and CD86 on neonatal CD103^+^ migDCs DCs isolated from mLNs. Results are expressed as mean ± SEM of *n* = 5 per age group. **p < 0.01, ***p < 0.001, ns, not significant, determined by repeated measures two-way ANOVA with Sidak *post-hoc* test.

Next, to examine trafficking of leukocytes, we euthanized animals 24 h after intranasal administration of DQ-OVA and isolated the draining lymph nodes (Figure 6A). In the lymph node single cell suspension, we gated lung migratory DCs as CD11c^+^, MHC-II^high^, and DQ-OVA^green+^ cells (Figure 6B) (33). Neonates compared to adults demonstrated lower basal percentages of both the cDC subsets, while mice receiving flagellin-adjuvanted DQ-OVA demonstrated higher trafficking of immunogenic migDCs (Figure 6C). Notably, flagellin also increased the percentage of adult CD11b^+^ cells in mLNs (Figure 6C). Moreover, flagellin upregulated the expression of co-stimulatory molecules on neonatal CD103^+^ DCs, while no significant differences were observed on the other subset studied (Figures 6D,E). These observations clearly show that TLR5 agonist adjuvantation mediated stimulation potentially activates the lung CD103^+^ and CD11b^+^ migDC subsets in neonates.

### Flagellin Regulates Phagosome Maturation and Antigen Degradation by Neonatal Lung CD103^+^ DCs

**Figure 7:**
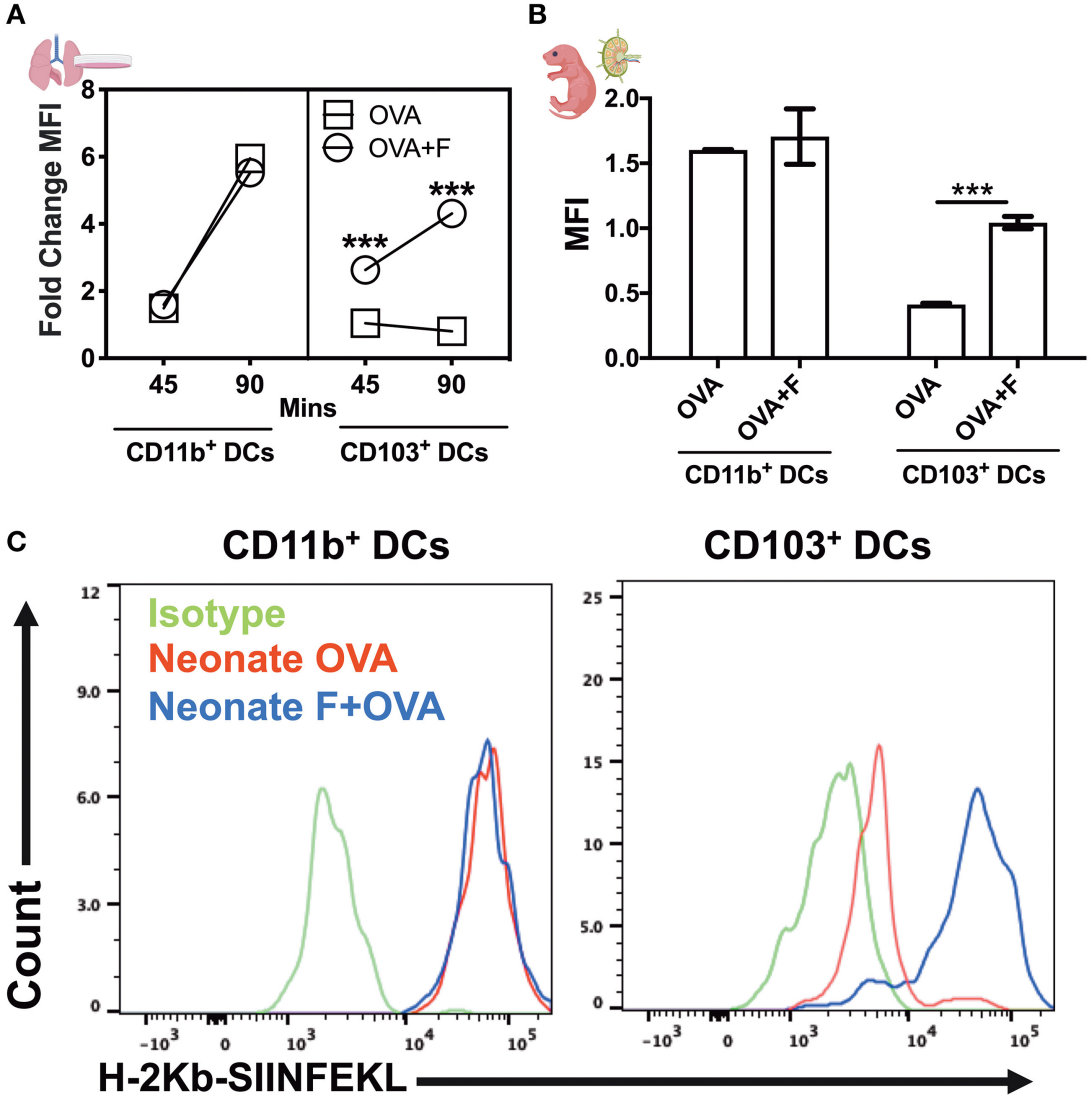
Flagellin enhances antigen cross presentation by neonatal lung CD103^+^ DCs. **(A)** DQ-OVA gives green fluorescence following uptake, and the co-emergence of a red signal reflects the accumulation of cleaved OVA peptides enabling assessment of cytoplasmic differences in organelle distribution after endocytosis. Stimulation of neonatal lung CD11c^+^ cells *in vitro* with flagellin measured the phagosome accumulation of DQ-OVA peptides in a time-dependent manner. **(B)** Antigen processing capacities are significantly increased by flagellin in isolated neonatal LN migDC subsets, as determined by the MFI ratio of OVA^green^ to OVA^red^ signal. **(C)** Anti-mouse MHC Class I antibody bound to SIINFEKL specifically reacts with ovalbumin-derived peptide SIINFEKL bound to H-2Kb of MHC class I, but not with unbound H-2Kb or H-2Kb bound with an irrelevant peptide. Draining lymph node CD103^+^ DC subset from the neonates show poor MHC-I presentation of OVA peptides (SIINFEKL), which was reversed in the flagellin adjuvanted group. *n* = 5 per age group. ***p < 0.001, ns, not significant, determined by repeated measures two-way ANOVA with Sidak *post-hoc* test.

By using DQ-OVA, we not only quantified cell trafficking but also characterized antigen uptake, degradation, and cytosolic distribution. DQ-OVA gives green fluorescence following proteolytic degradation in endosomes, and the co-emergence of a red signal reflects the accumulation of cleaved OVA peptides enabling assessment of cytoplasmic differences in organelle distribution after endocytosis. First, we stimulated lung CD11c^+^ cells *in vitro* with flagellin and measured the phagosome accumulation of DQ-OVA peptides (Figure 7A). While there was no overall difference in DQ-OVA uptake and degradation by DCs from neonates and adults (Figure S2), we noted the reduction (or a failure to exhibit an increase) in DQ-OVA^red^ signal in neonates CD103^+^ DCs in a time dependent manner, which was corrected by flagellin stimulation (Figure 7A, *P* < 0.001). In line with our *in vitro* studies, we explored this process *in vivo* using our LN migration model. Once again, the flagellin adjuvanted group demonstrated increased OVA^red^ signal, but only in neonatal, and not adult (not shown), CD103^pos^ DCs (Figure 7B, *P* < 0.001). Notably, we did not observe any significance downregulation or impaired phagosome maturation in either adult CD103^+^ or CD11b^+^ DCs as compared to their neonatal counterparts subsets (not shown). As slow phagosome maturation is a key step for antigen cross-presentation, these observations suggest that flagellin adjuvant in neonates, but not necessarily adult mice, might enhance a mucosal resident cytotoxic T cell response. These findings were further strengthened by the observations that the CD103^+^ DC subset from neonatal mice show poor MHC-I presentation of OVA peptides (i.e., SIINFEKL), which was potentially upregulated in the flagellin adjuvanted group in the draining lymph nodes (Figure 7C). Together, these studies show that TLR5 signaling plays an important role in the maturation, migration and antigen cross presentation of CD103^+^ DC subsets which possibly resonate with recent findings which highlight the adjuvanticity of TLR5 ligands via the mucosal route (34–36).

## Discussion

Respiratory infection is a major cause of infant mortality, whose susceptibility appears to be due in part to immunological differences in the mucosal compartment (2). Neonatal mucosal barriers are bombarded by environmental, nutritional, microbial, and pathogenic exposures after birth (37). Study of mucosal immune ontogeny may allow us to better understand the establishment of host-microbe homeostasis and responses to pathogens and vaccines. Indeed, the majority of global immunization schedules are focused on the pediatric age group. However, newborns display distinct immune responses, leaving them vulnerable to infections and impairing responses to some vaccines via the slow initiation, deceased magnitude of immunogenicity, reduced persistence of functional Abs, and cell-mediated responses (23). In addition, infectious pathogenesis in neonates might significantly differ from that of older children and adults (23). Vaccine development pipelines typically do not rationally tailor formulations (adjuvants, delivery systems, etc.) for use in early life, but studies such as ours suggest that it is important to take into account early life immune ontogeny to optimize vaccine efficacy (21, 38, 39). For example, early life vaccination against intracellular pathogens has proven difficult (40). Neonates and infants therefore may require different therapeutic vaccine approaches as compared to the established regimen applied to adults.

Innate immune response in early life, especially at the mucosal site is important not only to provide the protection against infections but also to activate adaptive immunity and generate a memory cell pool. Neonates typically demonstrate polarized activation of innate immunity with strong Th2 but limited Th1 responses to most stimuli, low interaction of migratory DCs with lymphocytes in the lymph nodes, and often-inefficient adaptive responses (32). In the present study, we numerically, phenotypically and functionally characterized the APCs isolated from neonatal and adult mouse lungs and monitored their response to different PRR stimuli. In the CD11c enriched cells, we identified mature APCs as MHC-II^high^, and four major APC precursors were gated based on the expression of Ly6C^+/−^ and CD11b^+/−^ in the MHC-II^low^ population. Compared to adult mice, neonatal mice demonstrated lower frequencies of CD103^+^ and CD11b^+^ migDC subsets, while the percentages of Ly6C^+^ precursor cells were abundant. Low percentages and qualitative differences in migDC subsets correlate with severity of RSV infection in the lungs of neonatal mice (32). However, the Ly6C^+^CD11b^+^ monocytic precursor population has not been fully explored for the transcriptional and functional characteristics to identify the molecular pathways, which can be targeted to generate mature APC and cDC populations. Interestingly, precursor cells are more proliferative and replenish DCs and maintain innate responses to microbes, while MHC-II^pos^ mature DCs are migratory and initiate adaptive immunity (41). After viral infections, precursor population depletion and loss of migratory receptor in MHCII^+^ cells is a mechanism of immune evasion (41, 42), a process that is incompletely characterized in early life. In this study, we focused mainly on mature cell phenotype and how can we overcome a hyporesponsive immune response in early life. Future studies should assess the impact of the high abundance of precursor cells, on infection or intranasal vaccination. Remarkably, we noted that neonatal mice demonstrated a lower percentage of type-I interferon secreting pDCs, while there were no differences noted in the antigen uptake capacities of all the APC subsets isolated from neonates and adults. These observations are consistent with prior reports that neonatal lung contains exceptionally low percentage of pDCs which were more abundant in the spleen, constituting ~40% of the mature APCs (43). Quantitative differences in lung migDC subsets in the lungs were also accompanied by phenotypic deficiencies of their maturation as they displayed the lower expression of maturation markers CD40, CD80, and CD86. Notably, adult and neonatal CD11c^pos^ cells demonstrated comparable cytokine production after stimulation with TLR5, RIG-I, and STING agonists, while there was no difference in the basal level of cytokine production. Based on these observations, we speculate that the low abundance or immature phenotype of migDCs and pDCs in the lung may contribute to the greater susceptibility to respiratory infection in early life (44).

To characterize the function of these APC subsets and identify potential innate pathways to overcome neonatal hyporesponsiveness, we stimulated CD11c-enriched cells with different PRR agonists and investigated the expression of maturation markers. Using *in vitro* bone marrow-derived DCs from neonates and adult, we previously identified a STING agonist (2′3′-cGAMP) as an effective adjuvant in early life (mice that were ~7 days old), that expanded GC B cells and subsequent antigen specific antibody titers, and IgG2c class switching as early as 42 days of life (25). However, the mucosal CD11c^+^ DCs from neonates and adults demonstrated distinct patterns of activation to the same stimuli, with flagellin the most active of the tested agonists *in vitro*. While responses to PRR agonists PAM3CSK4, PAM2CSK4, and PolyI:C were lower in both ages, we noted that the MPLA, CL075, CpG, and STING induced robust activation of lung DCs from mice lungs. Adjuvant-driven activation of migDC is relevant as these cells not only produce cytokines and induce leukocyte chemotaxis, but also migrate to draining lymph nodes and initiate adaptive immunity.

Among the stimuli studied, TLR5 agonist flagellin activated neonatal migDCs more robustly and upregulated the expression of lymph node homing receptor CCR7 on neonatal migDCs. These findings corroborate many studies which highlight flagellin as a potent mucosal adjuvant and highlight its role in the cytokine induction, leukocyte chemotaxis, cellular interactions, and the generation of protective innate immunity in the mucosal compartment (45, 46). To track migDC mobilization to lymph nodes, we administered DQ-OVA intranasally to neonates and adult mice. DQ-OVA not only acts as an antigen, but is also a very useful tool to study the antigen processing and presentation *in vivo*. While unstimulated DCs from neonates showed poor mobilization from the lung to lymph nodes in response to DQ-OVA antigen, flagellin co-administration strongly potentiated the migration of both the subsets and also upregulated the expression of CD80 and CD86 on CD103^+^ DC in the lymph nodes. These observations further strengthen our findings and highlight flagellin as a potent mucosal adjuvant in our early life intranasal vaccination model.

Another intriguing finding of our study is the differences in the lysosomal processing of antigens at an early age. DQ-OVA proteolytic cleavage emits the green fluorescence in the endosome, while the accumulation of OVA peptides in the perivascular compartment generates a strong red signal. While there were no differences in antigen uptake across both subsets, neonatal CD103^+^ DCs showed very rapid cleavage and clearance of DQ-OVA and significantly lower signal in the red channel, which signifies poor antigen cross presentation *in vivo*. We also examined the antigen processing capacities of isolated lung migDC subsets, and observed a striking difference in the DQ-OVA processing which signify the endosomal defects in the cross-presenting machinery of CD103^+^ DCs. However, flagellin stimulation revived phagosomal maturation and overcame the slow perivascular accumulation of antigens. We also studied OVA peptide presentation on MHC using anti-SIINFEKL antibodies specific for SIINFEKL bound to H-2Kb of MHC class I *in vivo*, and demonstrated that flagellin co-administration significantly increased antigen presentation on MHC-I. These findings suggest that TLR5 signaling in CD103^+^ DCs plays an important role in the priming of antigen cross-presentation. However, there is also evidence that flagellin-OVA fusion proteins may promote antigen cross-presentation independently of TLR5 or MyD88 signaling, by facilitating antigen processing, presumably dependent upon the maturation stage of DCs (47).

While dealing with the continual process of immune education during postnatal development, early life lung-resident immune cells are faced with the distinct challenge of reacting to pathogenic and non-pathogenic exposures in a manner that protects the organ's critical gas exchange machinery. This “neonatal window of opportunity” is such that early life priming can set the stage for life long host-microbial interaction and immune homeostasis (48–51). Additionally, composition of the early life microbiome may ultimately affect vaccine efficacy (52). For example, several theories are emerging regarding immune ontogeny and exposure during infancy in relation to immune perturbations such as pathogen exposure and vaccination. These include (a) the “hygiene hypothesis” (53), (b) the “appropriate exposure hypothesis” (54), (c) the “founder hypothesis” (55), and (d) “trained immunity” and “local innate immune memory” in the lung (56, 57). Interestingly, human cDC1 and cDC2 subset distribution is a function of tissue site and basal cDC2's preferentially exhibit maturation and migration phenotypes in mucosal-draining lymph nodes of adults and elders (58). However, this high cDC2:cDC1 ratio is (a) largely confined to intestinal sites in early life, and (b) cDC2 maturation dominance only emerges in the lung in later infancy (~3 months of life) and is maintained throughout life thereafter. Our studies provide further support for an ontologically driven cDC2:cDC1 ratio in the lung, and refine it to specially highlight a cDC1 dominance in the neonatal period that is supplanted by cDC2 subsets in infancy.

Overall, our study features several strengths, including (a) the immunophenotypic characterization of murine five neonatal lung APC subsets, by age; (b) a systematic screening of PRR agonists for activity toward neonatal lung DCs; and (c) while flagellin has been proposed as a promising adjuvant by IM route in NHP infant animals (59), or via the mucosal route in adult mice (60), our study is the first to combine identification of flagellin as an adjuvant active toward neonatal leukocytes *in vitro* and neonatal animals via a mucosal route *in vivo*, (d) distinct from prior investigations of how live infection of neonatal mice with *Staphylococcus aureus* or RSV effect migDCs responses, our study highlights the potential benefit of understating the unique efficacy of well-defined PRR stimuli/adjuvants (i.e., flagellin) enhance lung APC. Together, this information is critically important for evaluating the potential utility of rationally designed vaccine formulations in enabling early life intranasal immunization. Along with its multiple strengths, our study also has limitations, including (a) the neonatal lung APC model represents a mix of cells isolated by mechanical and enzyme treatment *in vitro* such that they may not fully reflect *in vivo* biology, (b) as a euthanizing agent we employed CO_2_ which can induce pulmonary hemorrhage in mice and activate lung APCs (61), and although we did not notice any substantial difference in neonatal and adult lung CD11c^pos^ for the production of IL-1β, we cannot rule out that changes in pH or oxygen tension in the lung may have impacted APC responses, (c) the potential effects of flagellin on migDC1 to lymph organs are intriguing but at this stage inferential, (d) although our studies demonstrated cDC1 maturation with flagellin adjuvanted intranasal ova vaccination, future functional studies (e.g., pathogen challenge) are required to assess the efficacy of this adjuvantation system, and (e) due to species specificity, results in mice may not accurately reflect those in humans.

To our knowledge, ours is the first study to functionally characterize mucosal APC subsets by age combined with evaluation of their responses to different PRR agonists, which can be used as mucosal adjuvants and/or potentiators of innate immunity to enhance resistance to respiratory infections in early life. We also highlight that the neonatal phase of murine development features qualitatively distinct CD103^+^ DCs that are relatively deficient in phagosomal maturation and cross-presenting machinery, which could be functionally enhanced via TLR5 stimulation. As there is an unmet medical need for robust mucosal adjuvants to protect vulnerable populations such as the very young, future studies should further address additional mechanistic differences in the endosomal compartment as well as the effect of flagellin adjuvantation on antigen immunogenicity *in vivo*, including potential protection from live microbial challenge.

## Data Availability Statement

The datasets generated for this study are available on request to the corresponding author.

## Ethics Statement

The animal study was reviewed and approved by Animal Care and Use Committee of Boston Children's Hospital and Harvard Medical School (protocol numbers 15-11-3011 and 16-02-3130).

## Author Contributions

PS, DD, and OL designed the study. PS conducted the experiments. PS and DD analyzed the data and wrote the manuscript. OL provided overall mentorship and assisted in writing the manuscript.

### Conflict of Interest

The authors declare that the research was conducted in the absence of any commercial or financial relationships that could be construed as a potential conflict of interest.

## Supplementary Material

Figure S1 Lung APCs from neonatal mice demonstrate comparable cytokine production to adult mice following stimulation with flagellin and 2′3′-cGAMP. **(A)** Summary of cytokine production after stimulation with different PRR agonists. **(B–E)** TLR5 agonist flagellin and the STING agonist 2′3′-cGAMP induced comparable cytokine production from both neonatal and adult APCs. *n* = 3 per age group, **p* < 0.05, ***p* < 0.01, ****p* < 0.001, ns, not significant, determined by repeated measures two-way ANOVA with Sidak *post-hoc* test.

Figure S2 migDC subsets from neonatal and adult mice demonstrate similar antigen uptake and endosomal processing. **(A,B)** CD11c enriched cells from neonatal and adult mouse lungs were stimulated with flagellin and were monitored for kinetics of DQ-OVA degradation. Cells were fixed and fluorescence measured in the green channel via flow cytometry. Mean of fluorescence intensities were compared for mice of different treatment groups.

Table S1 Antibodies used for flow cytometry study of APC subsets.

## Abbreviations

MTT: 3-(4,5- dimethylthiazol-2-yl)-2,5-diphenyltetrazolium bromide
AMs: alveolar macrophages
ANOVA: Analysis of variance
Kb: anti-mouse MHC class I molecule
Abs: antibodies
APCs: antigen presenting cells
BCH: Boston Children's Hospital
CD: cluster of differentiation
cDCs: conventional DCs
(2′3′-cGAMP): cyclic [G(2′,5′)pA(3′,5′)p]
DOL: day of life
DQ-OVA: DQ™ ovalbumin
EU: endotoxin units
EDTA: ethylenediaminetetraacetic acid
Ig: immunoglobulin
MACS: magnetic activated cell sorting
MHC: major histocompatibility complex
mLNs: mediastinal lymph nodes
migDCs: migratory DC
mAb: monoclonal Ab
MPLA: monophosphoryl Lipid A
ODNs: oligonucleotides
PAM2CSK4: Pam2CysSerLys4
PAM3CSK4: Pam3CysSerLys4
PRR: pattern recognition receptor
Kb-SIINFEKL: peptide SIINFEKL Abs
PBS: phosphate-Buffered Saline
pDCs: plasmacytoid DCs
PDCA-1: plasmacytoid dendritic cell antigen-1
RBC: red blood cell
RSV: respiratory syncytial virus
FLA-ST: *Salmonella typhimurium* flagellin
TLR: Toll-like receptor.
