# Supplementary material for: The TLR5 Agonist Flagellin Shapes Phenotypical and Functional Activation of Lung Mucosal Antigen Presenting Cells in Neonatal Mice
Source: Front Immunol. 2020 Feb 18;11:171. doi: 10.3389/fimmu.2020.00171 (PMC7039933; doi:10.3389/fimmu.2020.00171)
Supplement: Figure S1 — Lung APCs from neonatal mice demonstrate comparable cytokine production to adult mice following stimulation with flagellin and 2′3′-cGAMP. (A) Summary of cytokine production after stimulation with different PRR agonists. (B–E) TLR5 agonist flagellin and the STING agonist 2′3′-cGAMP induced comparable cytokine production from both neonatal and adult APCs. n = 3 per age group, *p < 0.05, **p < 0.01, ***p < 0.001, ns, not significant, determined by repeated measures two-way ANOVA with Sidak post-hoc test. [file Data_Sheet_1.PDF]

Supplementary Material

Table SI. Antibodies used for flow cytometry study of APC subsets

Anti-mouse Abs	Fluorochrome	Clone	Cat. No	Company
CD11c	PerCp	N418	117326	Biolegend
F4/80	Pacific Blue	BM8	123124	Biolegend
CD11b	PE-Cy7	M1/70	101216	Biolegend
CD103	APC	2E7	121414	Biolegend
PDCA1	BV510	927	747607	BC Biosciences
MHCII	eFluor780	M5/114.15.2	107628	Biolegend
Ly6C	PE	HK1.4	128008	Biolegend
CD80	FITC	16-10A1	104706	Biolegend
CD86	PE	GL-1	105008	Biolegend
CD40	FITC	HM40-3	102906	Biolegend

Figure S1

A

Average	In vitro stimulation	IL-12p70 A	IL-12p70 N	IL-1β A	IL-1β N	TNF A	TNF N	IL-6 A	IL-6 N
TLR2/1	PAM3CSK4	99	119	585	606	4511	7232	8390	14375
TLR2/6	PAM2CSK4	109	92	483	406	5346	4536	11249	9032
TLR3	Poly (I:C)	128	109	486	407	5382	4512	14202	8903
TLR4	MPLA	210	124	800	603	9097	8122	18098	8934
TLR5	Flagellin	214	163	882	796	9940	9000	19914	18117
TLR8/7	CL075	204	104	806	473	8819	5383	18288	10847
TLR9	CpG ODN	191	97	781	480	9060	5278	17962	10905
NOD1	C12 DAP	99	102	403	388	4474	4501	8910	8992
NOD2	L18-MDP	127	104	726	395	8122	4546	13547	9055
Mincle	TDB	101	88	400	400	4437	4562	9394	9037
RIG-I	ppp-dsRNA	196	163	801	640	8828	7235	17989	14655
STING	cGAMP	205	141	790	845	9162	9451	17973	18978

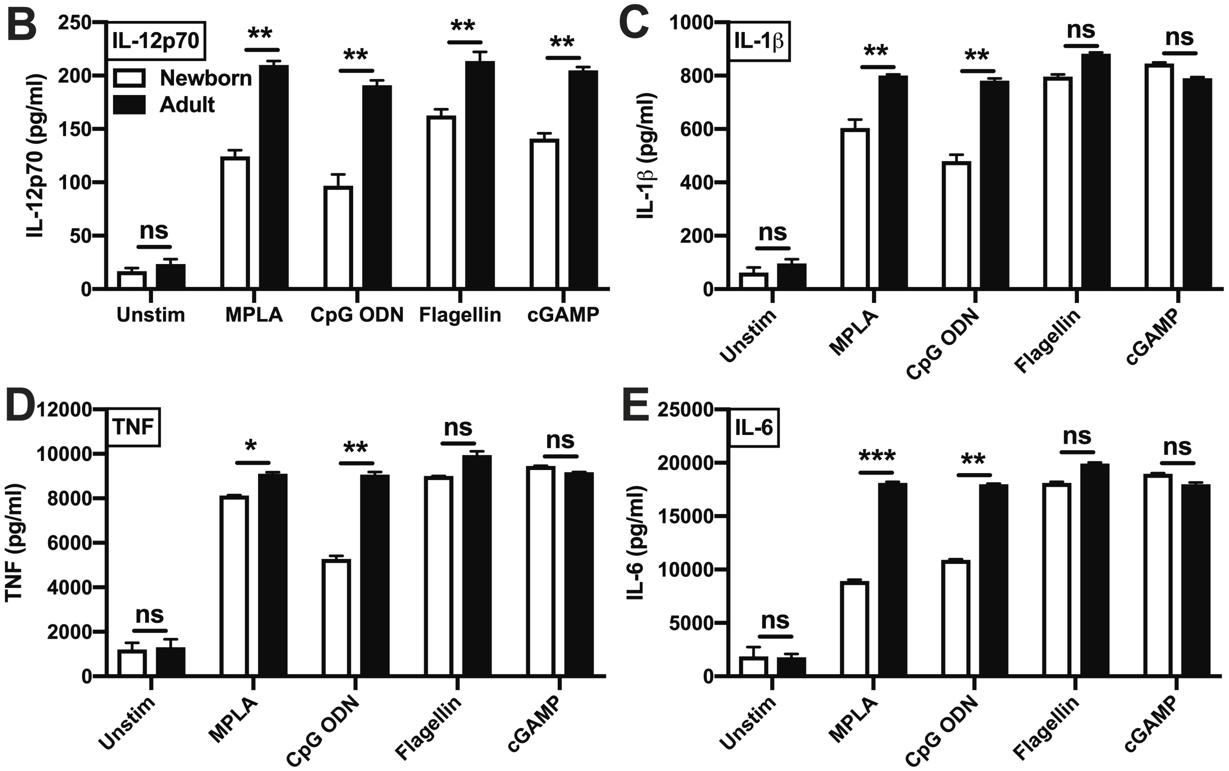

Figure S2

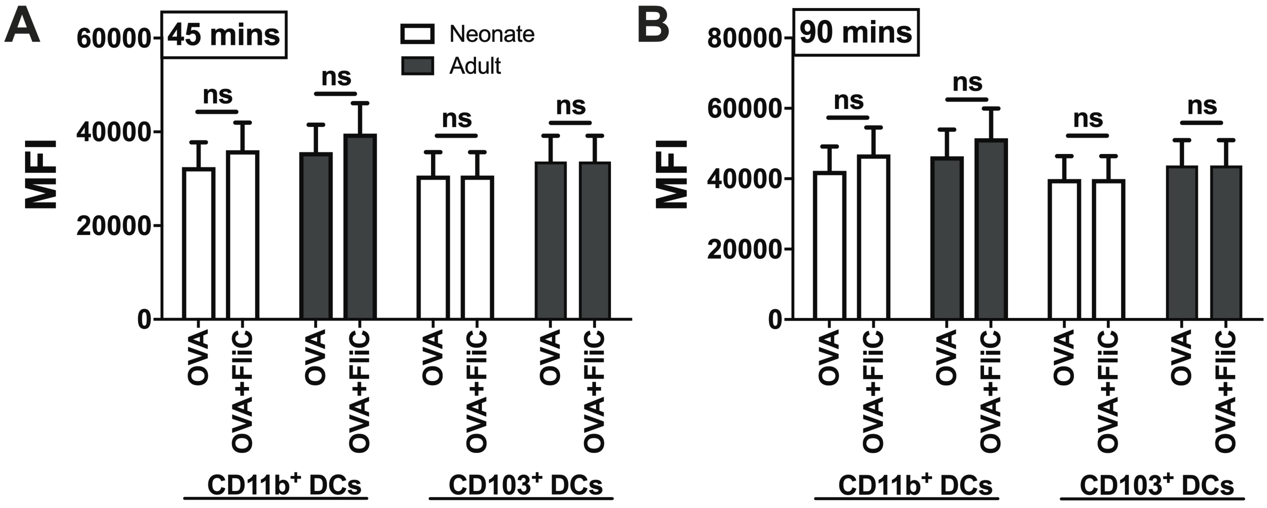
A
45 mins
Neonate
Adult
MFI
60000
40000
20000
0
ns
OVA
OVA+FliC
CD11b+ DCs
CD103+ DCs
B
90 mins
80000
60000
40000
20000
0
